# *Brucella abortus* Encodes an Active Rhomboid Protease: Proteome Response after *Rhomboid* Gene Deletion

**DOI:** 10.3390/microorganisms10010114

**Published:** 2022-01-06

**Authors:** María Inés Marchesini, Ansgar Poetsch, Leticia Soledad Guidolín, Diego J. Comerci

**Affiliations:** 1 Instituto de Investigaciones Biotecnológicas “Dr. Rodolfo A. Ugalde”, IIB-UNSAM-CONICET, Universidad Nacional de San Martín, San Martín, Buenos Aires 1650, Argentina; sguidolin@iib.unsam.edu.ar; 2 College of Marine Life Sciences, Ocean University of China, Qingdao 266100, China; ansgar.poetsch@rub.de; 3 Center for Marine and Molecular Biotechnology, Qingdao National Laboratory for Marine Science and Technology (QNLM), Qingdao 266237, China; 4 Department of Plant Biochemistry, Ruhr University Bochum, 44803 Bochum, Germany; 5 Centro Atómico Ezeiza, Grupo Pecuario, Comisión Nacional de Energía Atómica, Buenos Aires 1804, Argentina

**Keywords:** *Brucella* *abortus*, rhomboid, protease, label-free proteomics

## Abstract

Rhomboids are intramembrane serine proteases highly conserved in the three domains of life. Their key roles in eukaryotes are well understood but their contribution to bacterial physiology is still poorly characterized. Here we demonstrate that *Brucella abortus*, the etiological agent of the zoonosis called brucellosis, encodes an active rhomboid protease capable of cleaving model heterologous substrates like *Drosophila melanogaster* Gurken and *Providencia stuartii* TatA. To address the impact of *rhomboid* deletion on *B. abortus* physiology, the proteomes of mutant and parental strains were compared by shotgun proteomics. About 50% of the *B. abortus* predicted proteome was identified by quantitative proteomics under two experimental conditions and 108 differentially represented proteins were detected. Membrane associated proteins that showed variations in concentration in the mutant were considered as potential rhomboid targets. This class included nitric oxide reductase subunit C NorC (Q2YJT6) and periplasmic protein LptC involved in LPS transport to the outer membrane (Q2YP16). Differences in secretory proteins were also addressed. Differentially represented proteins included a putative lytic murein transglycosylase (Q2YIT4), nitrous-oxide reductase NosZ (Q2YJW2) and high oxygen affinity Cbb3-type cytochrome c oxidase subunit (Q2YM85). Deletion of *rhomboid* had no obvious effect in *B. abortus* virulence. However, *rhomboid* overexpression had a negative impact on growth under static conditions, suggesting an effect on denitrification enzymes and/or high oxygen affinity cytochrome c oxidase required for growth in low oxygen tension conditions.

## 1. Introduction

Rhomboid proteases are a superfamily of intramembrane proteins highly widespread and conserved in the three domains of life. Rhomboid active sites are embedded in the membranes, where they cleave their substrates in or close to the transmembrane domains (TMDs). In eukaryotes, they have been implicated in a variety of processes including epidermal growth factor signaling in *Drosophila melanogaster* [1], lipid metabolism [2], energy production [3], chloroplast development [4], apoptosis regulation [5], endoplasmic reticulum (ER) protein trafficking [6] and surface antigen shedding in the apicomplexan parasites [7].

In bacteria, rhomboid proteases are highly conserved and ubiquitous, suggesting a relevant role in physiology [8,9]. However, the relevance and function of rhomboid proteases in bacteria remain poorly investigated. In *Providencia stuartii*, the rhomboid protease AarA cleaves an N-terminal fragment of TatA, a membrane-bound component of the twin-arginine translocase secretion apparatus. Processing of TatA activates the translocation process, allowing the export of an unknown quorum sensing signal [10]. In *Mycobacteria*, rhomboid deletion mutants display altered colony morphology, impaired biofilm formation and increased antibiotic sensitivity [11]. In the archaeon *Haloferax volcanii*, a rhomboid protease is involved in protein glycosylation of the S-layer [12,13], but the implicated molecular mechanism has not been uncovered yet. The best functionally and structurally-characterized is *Escherichia coli* rhomboid protease, GlpG [14,15,16]. A recent study links the survival of a pathogenic strain in the mouse gut to the function of GlpG [17], yet the mechanism has not been elucidated. In Gram-positive bacteria, it was postulated that YqgP plays a role in cell division and may be required for glucose export [18]. In addition, it was recently demonstrated that *Bacillus subtilis* YqgP cleaves the high-affinity magnesium transporter MgtE and that YqgP interacts with the membrane-bound metalloprotease FtsH in order to maintain bacterial magnesium homeostasis [19]. Another recent study identified a new role for *Shigella sonnei* GlpG and Rhom7, which are involved in quality control of membrane orphan proteins of respiratory complexes in this Gramnegative bacterium [20].

*Brucella* spp. are intracellular pathogenic bacteria responsible for brucellosis, a chronic and debilitating zoonotic disease. Brucellosis manifestations include sterility and abortion in animals, and non-specific clinical signs like undulating fever, joint pain and fatigue in humans. Consumption of contaminated dairy products or contact with infected animals are the main routes of infection in humans. Brucellosis remains endemic in most of the developing countries with significant impact on economy and on human and animal health [21].

*Brucella* pathogenesis relies on its ability to replicate in various mammalian cell types [22]. At the onset of the infection, *Brucella* infects and replicates inside phagocytic cells such as macrophages and dendritic cells. Next, bacteria disseminate to placental trophoblasts (in pregnant females), and to organs of the reproductive tract and the mononuclear phagocyte system to establish a persistent infection in the host [23,24].

The role of membrane proteases in *Brucella* physiology is poorly characterized. Lon is the most studied membrane-residing protease of *Brucella*. Several roles have been demonstrated for this protease: it is involved in the modulation of macrophage cytokine response at early infection stages [25]; it functions as a stress response protease [26]; and it has a regulatory role recently identified by comparative RNA-seq analysis [27]. Stress-response proteases have been better characterized. *B. abortus* HtrA is a periplasmic stress-response protease involved in resistance to hydrogen peroxide and to the antibiotic puromycin [28]. In *B. suis,* ClpA is involved in bacterial clearance from infected BALB/c mice and in temperature-dependent growth regulation [29]. ClpB has also been characterized as a stress-response protease involved in sensitivity to ethanol stress, high temperature, and acidic pH in *B. suis.* [30].

To explore the biological role of rhomboid in *Brucella*, we demonstrate that it is an active protease and then constructed a deletion mutant in *B. abortus*. In this work, a quantitative high-throughput comparative proteomics approach was applied to understand the importance of rhomboid in *B. abortus* physiology and to identify putative substrates of this intramembrane protease.

## 2. Materials and Methods

### 2.1. Bacterial Strains and Growth Conditions

Bacterial strains and plasmids used in this study are listed in Table 1. 

*B. abortus* strains were inoculated in tryptic soy agar (TSA) or in tryptic soy broth (TSB). Liquid cultures and plates were incubated at 37 °C, ON (200 rpm) or for 72 h, respectively. All work with live *B*. *abortus* was performed in a biosafety level 3 laboratory facility. *E. coli* strains were grown in Luria Broth (LB), at 37 °C ON. Antibiotics were added to the indicated concentrations: 50 μg/mL kanamycin, 5 μg/mL nalidixic acid, 50 μg/mL ampicillin (for *B. abortus* strains) and 100 μg/mL ampicillin (for *E. coli* strains). 

### 2.2. Construction of B. abortus ΔrhoX 

To obtain the *rhomboid* mutant strain by unmarked gene deletion, the regions flanking the *rhomboid* gene (*bab1_1275*) were amplified and ligated using recombinant PCR technique [36]. The primers used for PCR amplification of the 500 bp upstream and downstream regions are Rho_UP_Fw CAGAATTCCGAGAAAGAAGAGGTCGCAG- Rho_compl_Rv cggcaggcgccccttACGGT and Rho_DW_Rv ATGGATCCTTCACAACGTCACCGATTGA- Rho_compl_Fw aaggggcgcctgccgATCCTGCGCCATTTCTGGCT, respectively (complementary sequences in lowercase). DNA fragments amplified by PCR were used in an overlapping PCR. The amplified fragments were ligated into pK18*mob-sacB* vector [32]. Plasmids were transformed in *E. coli* S17λ*pir* and transferred to *B. abortus* 2308 by biparental mating [37]. After selection and counterselection of double recombination events, gene deletion was confirmed by PCR. 

### 2.3. Plasmid Constructions

Genetic complementation of the mutant strains was achieved by expression of C-terminal 3xFlag-tagged versions of rhomboid protein from plasmid pBBR1-MCS4-3xFlag (pLF) [34]. DNA fragments coding for rhomboid were amplified using FwRho CAGGATCCCTTCCGAAAGACAAGAGAGAG and RvRho GCACTAGTTCGCCTCAAAACGGCAT primers containing BamHI and SpeI sites, respectively. The PCR product was digested with BamHI and SpeI, and the resulting fragment cloned into the same sites of pLF to generate in-frame fusions to the 3xFLAG epitope under *lac* promoter control. The resulting construction, designated pLF_*rho*, was introduced in *B. abortus rhomboid* mutant by biparental mating.

Plasmid pLF_*rho* S153A was obtained by site-directed mutagenesis following the instructions provided by the manufacturers (Quick Change^R^ Site Directed Mutagenesis, Stratagene). Briefly, primers Rho (Ser153/Ala) Fw CGCTGGTGGGTGCGGCGGGCGCGATTTCG and Rho (Ser153/Ala) Rv CCCGAAATCGCGCCCGCCGCACCCACCAG were used in PCR amplification using pLF_*rho* as a template. PCR products obtained were transformed into *E. coli* K12-DH5α F’lq (Kan^R^) and colonies were cultured to purify plasmidic DNA. Plasmids were sequenced to detect the point mutation Ser (TCG) to Ala (GCG). The plasmid obtained pLF_*rho* S153A was transferred to *B. abortus* by biparental mating. 

The plasmid encoding the rhomboid fusion to enhanced yellow fluorescent protein (EYFP) was obtained as follows: *rhomboid* gene was amplified using primers FwRho CAGGATCCCTTCCGAAAGACAAGAGAGAG and RvRho GCACTAGTTCGCCTCAAAACGGCAT, containing BamHI and SpeI sites, respectively. The PCR product was digested with BamHI and SpeI, and the resulting fragment cloned into the same sites of pTrc-EYFP [35], yielding pTrc-*rho*_EYFP. For microscopy analysis, the indicated plasmids were introduced into *B. abortus* 2308 by biparental mating using the *E. coli* S17-λpir strain.

*Gurken* and *tatA* chimera genes were amplified by PCR from pKS505 and pKS508 [14], using primers pMalp2E_Fw: GAATTCTGACGATGACAAGGGGGCGGG and pMalp2E_Rv: GGATCCGAATAATTTTATCGCTCATTC, containing EcoRI and BamHI restriction sites, respectively. The PCR products were digested and cloned in pBBR1-MCS2 plasmid, yielding pBBR2-*gurken* and pBBR2-*tatA,* respectively. 

### 2.4. Microscopy Analysis

Exponential-phase cultures of *B. abortus* 2308 expressing *rhomboid* gene as a fusion to EYFP were placed on a microscope slide layered with a pad of 1% agarose in phosphate buffered saline (PBS) as described in [35]. Samples were examined on an IX81 microscope with an Olympus FV1000 confocal module (60× PLAPO objective, numerical aperture [NA] of 1.42). Images were processed with the Image J program (NIH, Bethesda, MD, USA).

### 2.5. Bioinformatic Analysis

Protein sequences were aligned using Clustal Omega Webservers [38]. The tool available at http://www.microbesonline.org/operons/ (accessed on 10 October 2021) was used for operon prediction. Protein localization was predicted using PSORTb [39]. Topology representation was performed withTMRPres2D [40]. Transmembrane helices were predicted using TMHMM Server v. 2.0 [41], HMMTOP [42,43] and Phobius [44]. Rhomboid three-dimensional (3D) structure was predicted using the I-TASSER server for protein structure and function prediction [45,46,47], which identifies structural templates from PDB and then assembles the template fragments into a full-length protein model.

### 2.6. Membrane and Periplasmic Fractionations and Western Blot Analysis

Total membrane preparation was performed as described in [35]. Periplasmic extractions were prepared as previously described [34]. Samples were processed for Western blot using an anti-FLAG M2 (Sigma) monoclonal antibody (1:5000), anti-GroEL (1:2000) and anti-OMP-2b (1:2000), kindly provided by Axel Cloeckaert, as primary antibodies, and goat anti-mouse conjugated to the infrared 680 or 800 fluorophores (LICOR) as secondary antibodies (1:20,000 dilution). Images were acquired with Odyssey image-scanner.

### 2.7. Substrate Preparation for Protease Assay

To prevent possible endogenous rhomboid cleavage, *E. coli* MG1655 Δ*glpEGR::kan* with the plasmids encoding the heterologous substrates [10] were used. Cell extracts were prepared according to [12]. 

### 2.8. In Vitro Protease Assay

*B. abortus* membranes were prepared as described in [35]. For in vitro protease assay, substrate preparations (100 μg) were incubated for 18 h with *B. abortus* membrane fractions (100 μg) and samples were further processed as described in [12]. 

### 2.9. Protease Assays in B. abortus

Substrate cleavage assays were performed under aerobic conditions. *B. abortus* strains harboring pBBR2-*gurken* or pBBR2-*tatA* plasmids were grown ON at 37 °C (250 RPM). Bacteria were then harvested for SDS–PAGE and Western blotting. Complemented strains carried simultaneously two plasmids (derived from pBBR1-MCS2 and pBBR1-MCS4) that were maintained with different antibiotic resistance.

### 2.10. SDS-PAGE and Western Blot 

Samples were boiled for 5 min before loading on SDS-PAGE gels. Proteins were transferred to nitrocellulose membranes and blots probed with anti-His antibody (Invitrogen). IR Dye secondary antibodies were used for detection on the Odyssey Infrared Imaging System. 

### 2.11. Cell Fractionation for MS Analysis

*B. abortus* strains were inoculated in TSB or RPMI (Gibco) supplemented with 3.78 mM (NH_4_)_2_SO_4,_ 6 μM FeSO_4_, 0.1 mM MgSO_4_, 6.32 mM Na_2_S_2_O_3_, 2 mM L-glutamine and 1% yeast extract [48]. Cells were pelleted (10,000× *g* 10 min, 4 °C) and culture medium re-centrifuged under the same conditions. Supernatants were filtered through a 0.22 μm nitrocellulose filter and precipitated with TCA 10% (*v*/*v*). To obtain membrane and cytoplasmic fractions, the cell pellets were resuspended in lysis buffer (50 mM HCl-Tris, 2 M NaCl (pH 7.5)) and disrupted by ultrasound (3 × 30 s, 80 W). Lysates were clarified (17,000 *g* for 20 min at 4 °C), and total membranes were pelleted by ultracentrifugation (100,000× *g* for 1 h at 4 °C). Cytoplasm and membrane proteins were precipitated ON with 100% acetone at −20 °C. All samples were resuspended in 1× SDS-PAGE loading buffer, incubated at 70 °C for 10 min and stored at −20 °C. 

### 2.12. SDS-PAGE

Thirty μg of each sample were loaded onto polyacrylamide 10% (*w*/*v*) gels. The gels were run at RT (20 mA) until samples were concentrated into one protein band in the separation gel. Proteins were visualized with a Coomassie brilliant blue (CBB-G250) stain [49].

### 2.13. In-Gel Tryptic Digestion

Excision of proteins from gel bands, then cutting into pieces of about 1 × 1 mm size, de-staining of gel was carried out as described [50]. Protein reduction and alkylation was done according to [51]. Tryptic peptide digest was obtained following procedure in [52]. Upon drying in SpeedVac, peptides were stored at −20 °C. For mass spectrometry, the peptides were re-dissolved in 20 µL 0.1% formic acid in LC-MS/MS grade water, with 2% (*v*/*v*) acetonitrile (buffer A). For analysis, 8 µL of peptides were injected. 

### 2.14. LC-MS

LC-MS setup consisting of an UPLC HSS T3 column and an UPLC Symmetry C18 trapping column, nanoAcquity, and LTQ Orbitrap Elite was described in [51]. For elution of the peptides a linear gradient of 0.1% formic acid in acetonitrile (buffer B) from 5 % to 30% within 165 min was applied, followed by 15 min linear gradient from 30% to 98% buffer B, then column equilibration for another 15 min (0–5 min: 2% buffer B; 5–10 min: 5% buffer B; 10–165 min: 30% buffer B; 165–180 min: 30–98% buffer B; 180–195 min: 2% buffer B). Settings of LTQ Orbitrap Elite system were according to [51]. 

### 2.15. Protein Identification and Quantification

Protein identification was performed by MaxQuant version 1.5.3.175 with Andromeda [53] and quantified with the LFQ algorithm [54] embedded in MaxQuant searching against the complete proteome database of *B. abortus* 2308 (Uniprot taxon identifier 359391). After quantification, intensities from the LFQ normalization were filtered and compared with Perseus (v1.5.8.5) [55], removing rows with fewer than two values under either condition. Search parameters were fully specific cleavage by trypsin with no more than two missed cleavages: carbamidomethylation of cysteine (static), oxidation of methionine (dynamic), glutamine (Gln) to pyro-glutamate (pyro-glu) at protein N-terminus. A protein was considered as significantly regulated at a *p* value ≤ 0.05 (Student’s *t*-test). Mass spectrometry raw data are included in Supplementary Table S1. 

### 2.16. Automated Growth Curves

Overnight TSB cultures of the different strains were diluted (1/100) in TSB to the same initial OD_600_. For shaking experiments, 100 µL of diluted cultures were plated at least by triplicate in 96-well microplates while 250 µL of diluted cultures were plated for static experiments, in order to obtain a low-oxygenated condition. Growth curve experiments were performed using a TECAN Infinite Sunrise microplate reader (Thermo). OD600 nm was measured every 30 min at 37 °C.

## 3. Results

### 3.1. Identification of a Rhomboid Protease in B. abortus 

**Figure 1:**
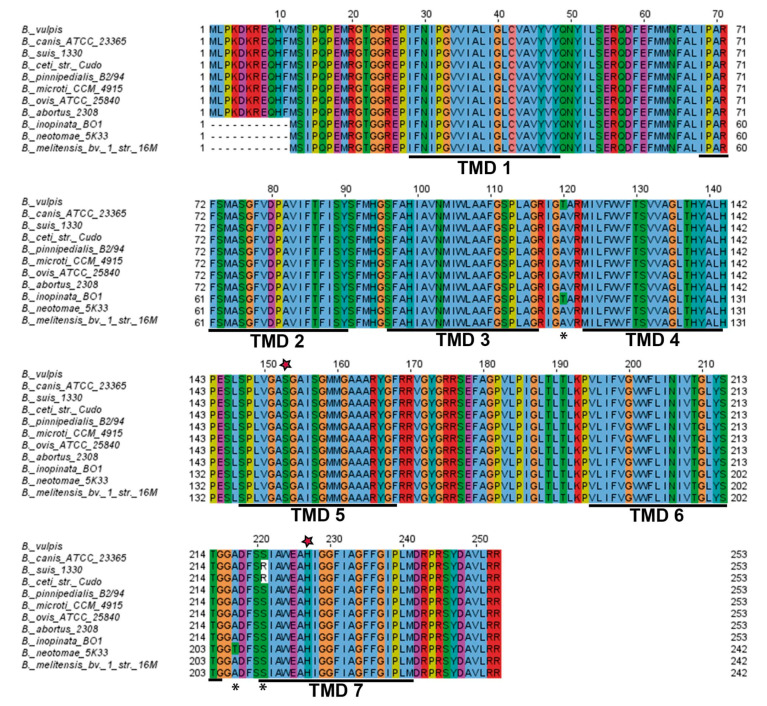
*Brucella* rhomboid protease. Multiple sequence alignment (Clustal omega) of annotated rhomboid proteases in *Brucella* species. TMD: transmembrane domain. Asterisks indicate amino acid changes and red stars in Ser(153) and His(227) (in *B. abortus*), indicate conservation of catalytic dyad residues in all species in TMD 5 and TMD 7, respectively.

To examine the occurrence of rhomboid in *Brucella*, the annotated sequenced genomes were manually verified using the BLAST program, available on the NCBI website. The in silico analysis showed that 292 annotated *Brucella* genomes encode a rhomboid-like protein. A unique genome organization at the rhomboid locus (bab1_1275 *in B. abortus* 2308) across the genus is observed, with many of the rhomboid surrounding genes conserved. Typically, flanking genes encode a cystathione β-synthase (CBS) domain-containing protein (bab1_1274 in *B. abortus* 2308) and a conserved hypothetical protein containing a PAS domain (bab1_1277 in *B. abortus* 2308). bab1_1274 and bab1_1275 are separated by 171 bp and they are predicted to be in the same operon (the probability is 0.628, according to the tool described in methods). Rhomboid proteases are highly conserved across the genus, except for a few amino acid substitutions in *B. suis*, *B. vulpis*, *B. canis* and *B. inopinata* (Figure 1). 

### 3.2. Localization of Rhomboid Protease in B. abortus

Four different topological classes for rhomboid-like proteins have been proposed [56]. The basic class consists of a six-transmembrane domain core. It is found in *E. coli* GlpG and some eukaryotic rhomboids such as *Saccharomyces cerevisiae* Rbd2 (YPL246C) [57,58,59]. The next class has an extra TMD fused to the C terminus and a variable N-terminal domain [1]. *Drosophila* Rhomboid-1 and many bacterial rhomboids have a 6 + 1 TMD structure. The third class contains a globular domain inserted into first loop and some modifications in the active site. Finally, the PARL-subfamily (Presenilin-Associated-Rhomboid-Like) has a predicted extra TMD fused to the N terminus of the rhomboid core, thus the catalytic residues are inserted in TMD5 and TMD7, instead of TMD4 and TMD6 in other rhomboids.

**Figure 2:**
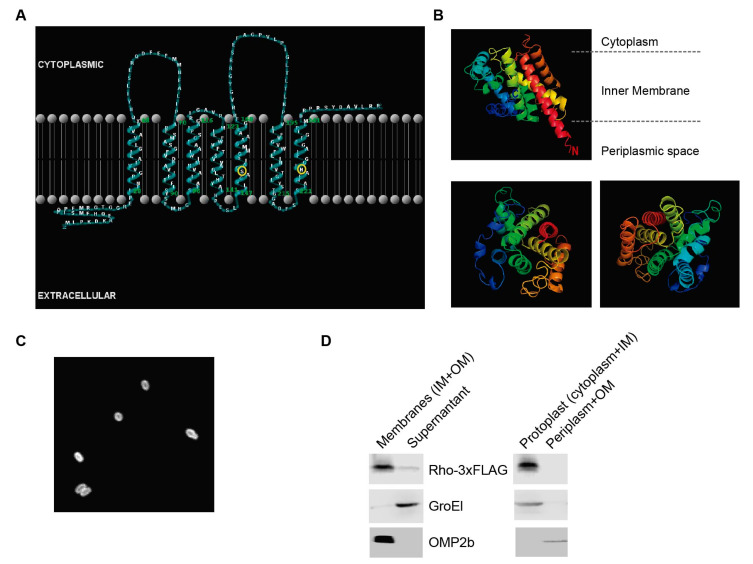
Topology of *B. abortus* rhomboid. (**A**) Topology model built with TMRPres2D. Rhomboid catalytic residues in TMD5 and TMD7 are circled (yellow). Rhomboid N-terminus is in the periplasm (extracellular) and C-terminus is cytoplasmic. (**B**) Predictive 3D structure of rhomboid by I-TSAAER. (**A**) Colored ribbons indicate transmembrane helices of rhomboid; N denotes the N-terminus; C denotes the C-terminus. Lower left: View from the periplasmic space; Lower right: view from the cytoplasm. Ribbons were colored using the rainbow color scheme implemented in PyMOL. (**C**) Fluorescence confocal microscopy images of *B. abortus* constitutively expressing Rho_ EYPF fusion protein. (**D**) Subcellular localization of rhomboid protease by Western blot analysis of the indicated fractions. *B. abortus* pLF_*rho* strain (FLAG staining); Omp2b, outer membrane protein 2b; GroEL, cytoplasmic chaperonin. OM: outer membrane; IM: inner membrane.

Based on several bioinformatic tools (HMMTOP, Phobius and TMHMM), *B. abortus* rhomboid is predicted to comprise seven TMDs (Figure 1 and Figure 2A). According to the topological classification, it belongs to the fourth class (1 + 6 TMD) together with the PARL subfamily of mitochondrial rhomboid proteases. The first sixteen to twenty-seven amino acids of the protein (depending on the species) are predicted to be in the extracellular space/periplasm, whereas the C-terminus is predicted to be cytoplasmic. The serine (GXSG) and histidine residues typical of the catalytic dyad of this protease family [60] are conserved in TMD5 and TMD7, respectively (Figure 1 and Figure 2A).

We further predicted the 3D structural model of rhomboid with the help of I-TASSER server. The crystallized synthetic protein *E. coli* GlpG (pdb 2iC8) [61] was ranked as the best template with a normalized Z-score of 2.14 for the alignment and 72% coverage. The C-score, which gives the confidence for the quality of the predicted models, is −0.83, and the TM-score is 0.61 ± 0.14 [45]). The predicted 3D structure (Figure 2B) contains seven transmembrane segments supporting *B. abortus* rhomboid as a transmembrane protein homologous to other proteins of the rhomboid family.

To ascertain rhomboid intramembrane localization, a *B. abortus* strain expressing rhomboid as a fusion to yellow fluorescent protein (EYFP) was obtained. As shown in Figure 2C, the fusion protein is localized to the bacterial membrane. Supporting this result, Western blot analysis of total membrane preparations of a strain expressing rhomboid as a fusion to 3xFLAG epitope, revealed a membrane association of the fusion protein, indicating that it is either an inner or an outer membrane-associated protein (Figure 2D). Further periplasmic extraction experiments showed that the protein is not associated to the periplasm or outer membrane, confirming its association to the inner membrane (Figure 2D).

### 3.3. B. abortus Rhomboid (RhoX) Is an Active Protease

**Figure 3:**
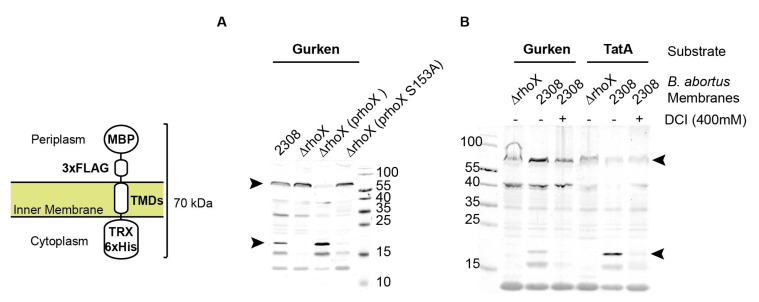
*B. abortus* membrane-associated rhomboid protease activity. (**A**) Cleavage of Gurken chimeric substrate by *B. abortus* solubilized membrane fractions of the indicated strains. Cell extracts of *E. coli* MG1655 ΔglpEGR::kan transformed with the plasmid for the expression of Gurken chimeric protein were incubated with the solubilized membranes. After incubation, samples were TCA-precipitated and used in SDS-PAGE and Western blot experiments with anti-His antibody. (**B**) Cleavage of Gurken and TatA chimeric substrates by *B. abortus* solubilized membrane fractions of the indicated strains in the presence or absence of 3,4-DCI inhibitor. Cell extracts of *E. coli* MG1655 ΔglpEGR::kan transformed with plasmids for the expression of Gurken or TatA chimeric protein were incubated with the solubilized membranes. After incubation, samples were TCA-precipitated and used in SDS-PAGE and Western blot with anti-His antibody. The bands corresponding to unprocessed and processed substrates are pointed out by arrow heads. Migration of molecular mass markers (kDa) is indicated in each blot. On the left, a schematic representation of the chimeric protein substrates is shown. 2308: wild type; Δ*rhoX*: *rhomboid* deletion mutant; Δ*rhoX* + p*rhoX*: *rhomboid* deletion mutant complemented with wild type rhomboid; Δ*rhoX* + p*rhoX*S153A: *rhomboid* deletion mutant complemented with rhomboid mutant S153A.

In the absence of a known substrate, the identification of active rhomboid proteases has been possible due to the capability of rhomboid proteases from evolutionarily divergent organisms to recognize and cleave a common set of substrates [1,14,62,63,64,65]. To determine if *B. abortus* rhomboid (RhoX hereafter) is an active protease, we tested the in vitro proteolysis of *D. melanogaster* Gurken and *P. stuartti* TatA, two model substrates for rhomboid proteases. These substrate analogues are chimeric proteins that consist of *E. coli* maltose binding protein (MBP) with a signal peptide (SP) at the N-terminus, followed by the TMD of Gurken or TatA protein, and a thioredoxin (Trx) domain and His tag at the C-terminus [14] (Figure 3). These model substrates was expressed in *E. coli* MG1655 ΔglpEGR::kan, and cell extracts were incubated with solubilized membranes from *B. abortus* wild type (2308) and a deletion mutant in *rhomboid* gene (Δ*rhoX*). As shown in Figure 3A,B, the model substrates were cleaved in the presence of *B. abortus* wild type membranes, releasing an ~18 kDa- His tagged C-terminal fragment, whereas the proteolytic activity was undetectable in the presence of *B. abortus* Δ*rhoX* membranes. The rhomboid activity was restored in membranes of the *B. abortus* Δ*rhoX* (p*rhoX*) complemented strain and only traces of activity were detected in membranes of *B. abortus* Δ*rhoX* (p*rhoX* S153A), complemented with a RhoX protein with a substitution of one of the residues of the catalytic dyad.

It was previously described that some rhomboid proteases are inhibited by the general serine protease inhibitor 3,4-dichloroisocoumarin (3,4-DCI) [1]. To test if *B. abortus* rhomboid activity is impaired by this inhibitor, we performed the in vitro assay in the presence of DCI with two rhomboid chimeric substrates: Gurken TMD and *P. stuartii* TatA TMD. Rhomboid activity towards Gurken and TatA chimeric substrates was completely abolished in the presence of 400 mM DCI (Figure 3B). 

**Figure 4:**
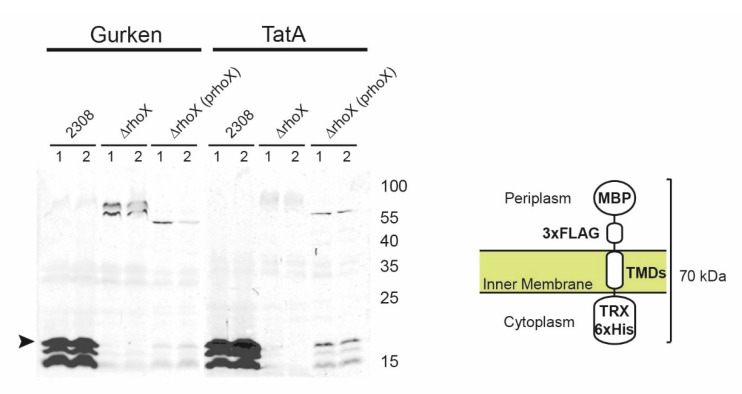
*B. abortus* rhomboid activity in vivo. Cleavage of Gurken and TatA chimeric substrates expressed by the indicated *B. abortus* strains (2308: wild type; Δ*rhoX*: *rhomboid* deletion mutant; Δ*rhoX* + p*rhoX*: *rhomboid* deletion mutant complemented with wild type rhomboid). Total protein extracts were analyzed by SDS-PAGE and Western blot with anti-His antibody (in duplicates). The bands corresponding to processed substrates are pointed out by an arrowhead. A schematic representation of the chimeric protein substrates is shown on the right.

To analyze if rhomboid proteolytic activity also occurs in vivo, we expressed two rhomboid chimeric substrates, Gurken and TatA, under constitutive promoters in *B. abortus*, and whole cell extracts were analyzed by Western blot. As shown in Figure 4, both substrates were cleaved when expressed in *B. abortus* 2308 but not in *B. abortus* Δ*rhoX* background. As shown in vitro, *B. abortus* Δ*rhoX* (p*rhoX*) complementation was able to restore the proteolytic activity in vivo. These results demonstrate that RhoX is active towards the model substrates in vivo.

### 3.4. Identification of Putative RhoX Substrates by Label-Free Quantitative Proteomics

**Figure 5:**
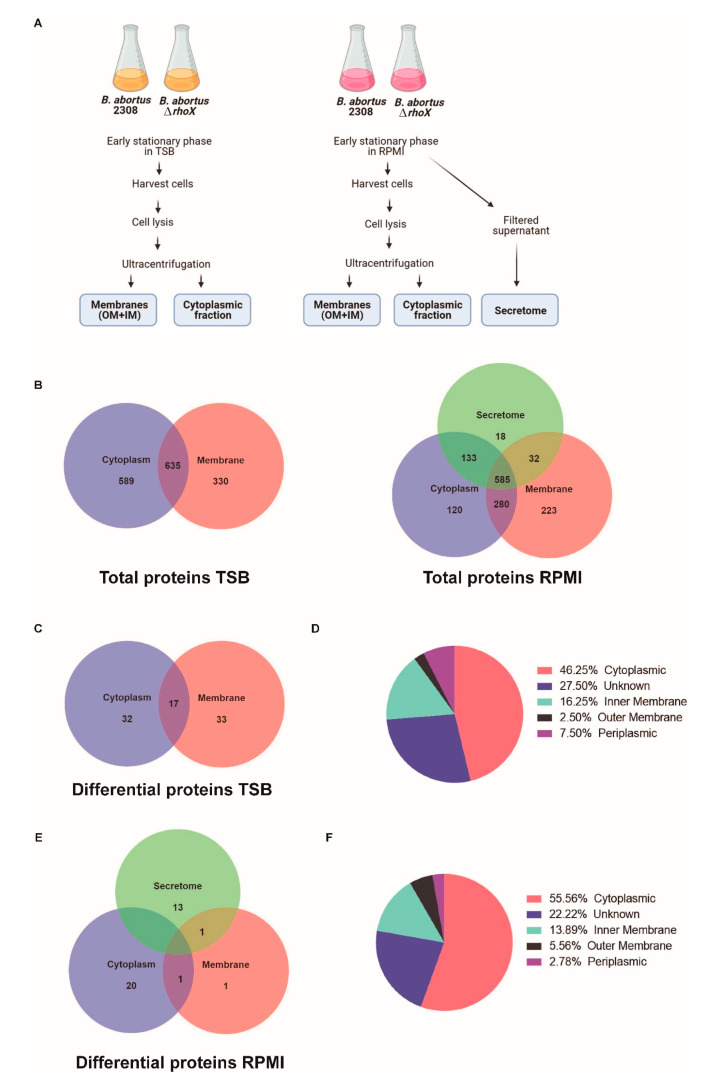
Quantitative proteomics screen to identify substrate repertoire of RhoX. Early stationary phase cultures of the indicated strains grown in TSB or in RPMI media were processed as described in methods, and the indicated subcellular fractions analyzed by LC-MS/MS (**A**)**.** Venn diagrams showing the total number of identified proteins in the indicated cellular fractions (left: TSB-grown bacteria; right: RPMI-grown bacteria) (**B**). Venn diagram showing the distribution of differentially represented proteins of TSB-grown bacteria (**C**). Proteins differentially represented (82) between 2308 and Δ*rhoX* (in cytoplasmic and membrane fractions of TSB-grown bacteria) were grouped by their predicted localization according to PSORTb (**D**)**.** Venn diagram showing the distribution of differentially represented proteins of RPMI-grown bacteria (**E**). Proteins differentially represented (36) between 2308 and Δ*rhoX* (in cytoplasmic, secretome and membrane fractions of RPMI-grown bacteria) were grouped by their predicted localization according to PSORTb (**F**).

Having demonstrated that RhoX is an active protease, we sought to identify putative substrates by a label-free quantitative proteomics approach. We also intended to address metabolic and signaling pathways altered in response to *rhomboid* deletion. To achieve this, the differential proteome map between the *B. abortus* wild type (2308) and *rhomboid* deletion mutant (Δ*rhoX*) was obtained. Two growth conditions were used to compare membrane, cytoplasmic and supernatant fractions of wild type and *rhomboid* mutant. Both strains were grown to early stationary phase (OD_600_ 1.8–1.9) in TSB rich medium (for membrane and cytoplasmic proteins) or in a defined minimal medium (supplemented RPMI) (for membrane, cytoplasmic and supernatant proteins) (Figure 5A). Proteins obtained from triplicate cultures of both strains were subjected to SDS-PAGE, digested with trypsin, and then analyzed by nano-LC-MS/MS as described in the Experimental Section.

Out of 3023 total proteins from the predicted *B. abortus* proteome, 1554 (51.41%) were identified from bacteria grown in TSB and 1391 (46.01%) from bacteria grown in RPMI, including both strains and all cell fractions. Venn diagrams indicating the number of identified proteins in each of the cellular fractions are shown in Figure 5B (left panel TSB-grown bacteria and right panel RPMI-grown bacteria). The protease RhoX was not detected in membrane fractions of *B. abortus* 2308. Indeed, integral membrane proteins containing several TMDs and/or hydrophobic stretches are barely detected by MS technology, as they are underrepresented in trypsin digestion products [66]. However, they can be identified with trypsin/chymotrypsin digestion [13].

### 3.5. Proteins Differentially Represented in ΔrhoX Mutant

Using the criteria described in M&M, a total of 82 and 36 proteins were differentially represented between 2308 parental strain and Δ*rhoX* mutant strain grown in TSB or RPMI media, respectively (Figure 5C,E). Supplementary Table S2 shows the proteins which evidenced significant increase, organized by growth media, strain and cellular fraction. Ten proteins were differentially represented both in TSB and in RPMI between wild type and Δ*rhoX* (Table 2). Out of 108 differential proteins, 22 were predicted to contain a signal peptide and 8 contain at least one transmembrane domain. 48.23% of the proteins that exhibited variation in their concentration were detected in membrane and secretome fractions, in accordance with RhoX exerting its role within the membrane. Accordingly, about 50% of the proteins were predicted to be in the inner membrane, outer membrane, periplasm or to have unknown localization as predicted by Psortb (Figure 5D,F).

**Figure 6:**
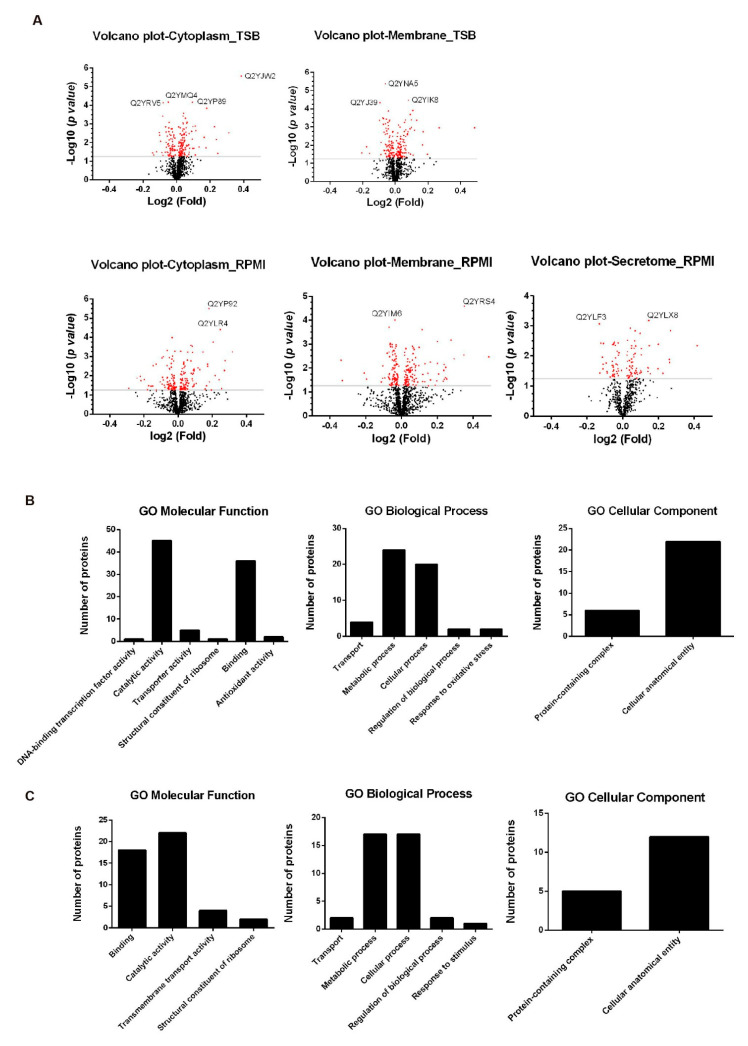
Graphical representation of quantitative proteomics data. (**A**) Total detected proteins from the indicated fractions of TSB- and RPMI-grown bacteria are ranked in volcano plots. Each data point represents a single unique protein identified in our proteomic analysis. Black indicates no significant change and red indicates significant (negative and positive) changes. Labels indicate proteins with lower statistical *p* values (higher significance). Gene ontology (GO) analysis of differentially represented proteins between wild type and Δ*rhoX* strains in TSB (**B**) and RPMI-grown bacteria (**C**). GO annotation results were divided into biological processes, cellular components, and molecular functions.

Volcano plots (Figure 6A) were generated to visualize protein abundance changes obtained in these experiments, by plotting −log10 (*p*-value) vs. log2 (fold-change) for the compared samples. Of the proteins meeting statistically significant criteria (*p* value ≤ 0.05), those with the lower *p* values (higher values of −log10 (*p*-value) were highlighted in the figures (Figure 6A). In the cytoplasmic fraction of TSB-grown bacteria, four proteins exhibited lower *p* values: Q2YJW2 (Nitrous-oxide reductase NosZ); Q2YP89 (Fumarylacetoacetate hydrolase); Q2YMQ4 (Periplasmic binding protein/LacI transcriptional regulator) and Q2YRV5 (immunogenic 31 KDa TRAP transporter solute receptor, TAXI family protein). In the membrane fraction of TSB-grown bacteria Q2YNA5 (DNA topoisomerase 4 subunit A), Q2YJ39 (ABC transporter: AAA ATPase) and Q2YIK8 (Heme transporter BhuA) evidenced the most significant difference in amount. In RPMI-grown bacteria, proteins meeting this criteria were Q2YLF3 (4-hydroxyproline epimerase) and Q2YLX8 (Ribose-phosphate pyrophosphokinase) in the secretome, Q2YP92 (Oxidoreductase, N-terminal) and Q2YLR4 (diaminopimelate epimerase) in the cytoplasm, and Q2YIM6 (PntA, NAD(P) transhydrogenase, alpha subunit) and Q2YRS4 (OmpW family protein) in the membrane. The differential proteins belonged to several functional classes (Figure 6B,C) and proteins involved in catalytic activity and binding were the most represented, suggesting that these processes are affected by RhoX. 

### 3.6. Proteins Differentially Represented in the Membrane Fraction: Potential Rhomboid Targets

Rhomboid activity may expose or release a fragment of the substrate, leading to changes in size, stability and/or location of the cleaved substrate. Most characterized substrates are single TMDs, type I or III proteins (N-terminus facing the extracellular side with or without a SP, respectively). Thus, single TMD proteins exhibiting accumulation or decrease can be considered putative targets of rhomboid. A total of 51 proteins were differentially represented in membrane fractions of TSB- and RPMI-grown bacteria (Table S2), comprising 47.22% of the total altered proteins. Six proteins identified as differential are predicted to have at least 1 TMD and seven contain signal peptides that likely target them to the cell surface. Proteins without predicted TMDs and/or signal peptides may be associated to the membrane via interactions with lipids or other proteins and might represent indirect targets of rhomboids. However, it cannot be ruled out that some of these proteins are contaminants from cytoplasmic origin. 

Q2YJT6 and Q2YP16 are integral membrane proteins (with single TMDs) that accumulated in the wild type membrane fraction. Q2YJT6 is nitric oxide reductase subunit C (NorC) and Q2YP16 is periplasmic protein LptC involved in LPS transport to the outer membrane.

### 3.7. Proteins Differentially Represented in the Secretome

Fourteen proteins were differentially represented in the secretome (Table S2). Out of these, seven are predicted secretory proteins, two are expected to be membranes associated via 1 TMD, and the remaining are likely soluble proteins. Vesicle transport (outer membrane vesicles) or cell lysis might explain the presence of proteins without a predicted SP in supernatants. Soluble proteins, with or without an SP, differentially represented may be a consequence of an indirect effect of the *rhomboid* deletion. Two integral membrane proteins with a single TMD were underrepresented in Δ*rhoX* culture media supernatants (Table S2). Q2YQA6 is a periplasmic protein, named EipA, involved in membrane integrity [67], and Q2YM85 is Cbb3-type cytochrome c oxidase subunit. 

### 3.8. Oxygen-Limiting Conditions and Rhomboid Protease

To explore the role of RhoX in *B. abortus*, we subjected Δ*rhoX* to multiple phenotypic assays including growth in rich/minimal media under aerobic or anaerobic conditions, resistance to detergents or to oxidative stress, intracellular replication in phagocytic and non-phagocytic cells, and virulence in mice. Deletion of *rhomboid* had no effect in these assays.

It has been recently described that *S. sonnei* rhomboid proteases GlpG and Rhom7 prevent protein aggregation of orphan proteins in the inner membrane [20]. They specifically target components of respiratory complexes. With that in mind, we noticed that many of the differentially represented proteins between *B. abortus* 2308 and Δ*rhoX* are associated to denitrification and oxidative phosphorylation complexes: Q2YJW2 nitrous-oxide reductase NosZ, Q2YJU8 copper-containing nitrite reductase, Q2YJT6 nitric oxide reductase subunit C (NorC), Q2YJY3 nitrate reductase subunit beta (NarH), Q2YJY4 nitrate reductase subunit alpha (NarG) and Q2YM85 (high oxygen affinity) Cbb3-type cytochrome c oxidase unit.

**Figure 7:**
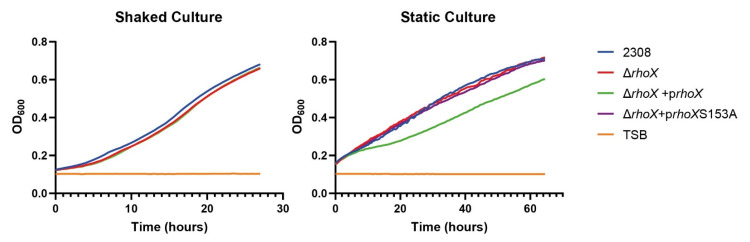
Growth curves under shake or static conditions. Overnight TSB cultures of the indicated strains were diluted in TSB to the same initial OD_600_. For shake conditions, 100 µL of diluted cultures were plated in p96 microplates while 250 µL of diluted cultures were plated for the static condition. Automated growth curves were performed in a multiwell plate reader, following the OD_600nm_ every 30 min at 37 °C on maximum agitation or without agitation. Non-inoculated medium (TSB) was included as a control. Figure shows a representative of 2 experiments with 3 to 5 biological replicates.

To investigate the biological role of RhoX we first addressed nitrate respiration in anaerobic conditions, but no differences could be detected among wild type, Δ*rhoX* and the complemented strains. Then, we evaluated bacterial replication under static or shake conditions in rich medium TSB. All strains behaved similarly under shake conditions (Figure 7 left panel). However, *rhomboid* mutant strain overexpressing wild type RhoX exhibited growth impairment under the static condition. Initial growth was similar, but after a few hours a metabolic change became evident as a diauxic curve. After adaptation, growth rate recovered to wild type levels, describing a linear performance as oxygen transference rate became the limiting factor (Figure 7 right panel). In contrast, complementation of the mutant with the mutated version of rhomboid (S153A) had no effect in the replication curve, indicating that adaptation to low oxygen conditions is affected by overexpression of a functional rhomboid protease.

## 4. Discussion

Here we identified and characterized *B. abortus* rhomboid protease. We found that it is catalytically active and cleaves model heterologous substrates like *D. melanogaster* Gurken and *P. stuartii* TatA in vitro and in vivo. This activity was abolished in the deletion mutant and restored after complementation with a wild type gene. Supporting these results, protease activity could be inhibited by (3,4-DCI), a general inhibitor of serine proteases including rhomboids [68,69].

Rhomboid proteases typically contain a six-transmembrane domain core with or without an extra TMD [8]. RhoX has an additional N-terminal TMD like mitochondrial PARL-like rhomboids. This might be related to the fact that mitochondria have an alphaproteobacterial ancestry [70]. In members of this subfamily, the GxSx and histidine of the catalytic dyad are shifted to TMD5 and TMD7, respectively. This topology differs from most of bacterial rhomboids, and it is not clear whether this difference infers evolutionary or functional significance, such as substrate recognition or regulation of proteolytic activity. Immunofluorescence and subcellular fractionation studies indicate that RhoX is an inner membrane protease. Cellular localization of the endogenous protein is yet to be done to confirm the localization determined for the EYFP and FLAG-tagged proteins.

Over the years, the search for rhomboid substrates in bacteria has remained challenging. A recent study has shed some light about the potential reasons [20]. The study shows that the substrates are only expressed during growth of *S. sonnei* in specific conditions and that cleavage can be impeded by the presence of partner proteins. More importantly, GlpG and Rhom7 rhomboid proteases selectively target non-functional proteins, hence their absence might not lead to noticeable phenotypes. Similar results were obtained after deletion of *B. abortus rhomboid*. None of the phenotypes evaluated were statistically different between wild type and Δ*rhoX.*

As a step towards defining the function of RhoX, we used a label-free quantitative proteomic approach. We analyzed differentially represented proteins in membrane, cytoplasmic and secreted fractions in wild type and Δ*rhoX* strains. This approach has been successful in identifying rhomboid substrates in *H. volcanii* [13], *Trichomonas vaginalis* [71] and *S. sonnei* [20], among others. 

About 50% of the predicted proteome was identified and 108 differentially represented proteins were detected. Of those showing the most significant variation (highlighted in volcano plots), three were predicted to contain signal peptide (Nitrous-oxide reductase NosZ, a 31 KDa immunogenic protein and OmpW family protein) and six were predicted to be either membrane-associated (Nitrous-oxide reductase (NosZ), ATP/GTP-binding site motif A (P-loop):ABC transporter: AAA ATPase, Heme transporter BhuA, NAD(P) transhydrogenase subunit alpha and OmpW family) or periplasmic (Periplasmic binding protein/LacI transcriptional regulator) proteins. The role of some of these proteins has been described: NosZ is the last reductase of the denitrification pathway and catalyzes N_2_O reduction to N_2_; OmpW family of small outer membrane proteins is widely distributed in Gram negative bacteria and has been associated to host-bacterial interactions in *Pseudomonas aeruginosa* and in many *Vibrio* species [72]. It could also participate in the transport of small molecules across the outer membrane [73,74]. Heme transporter BhuA is required to maintain a chronic spleen infection in BALB/c mice experimentally infected with *B. abortus* [75].

A high proportion of differentially represented proteins belonged to the membrane fraction of both TSB an RPMI-grown bacteria. Among these, of special interest are proteins with SP or single TMDs: transporters of branched amino acids (Leu/Ile/Val), a 31 kDa immunogenic protein, nitric oxide reductase subunit C (NorC), periplasmic protein LptC, secretion protein HlyD and OmpW family protein. The 31 kDa immunogenic protein is also known as BCSP-31. It is a *Brucella* surface antigen largely used in diagnostics of brucellosis [76]. NorC is a membrane-anchored c-type cytochrome catalyzing the reduction of NO to N_2_O. LptC is a component of the LPS transport machinery [77]. HlyD is the membrane fusion protein (MFP) component of type I secretion systems [78].

Since rhomboid substrates identified so far are integral membrane proteins, differentially represented proteins identified in cytoplasmic fractions are probably a result of yet unidentified gene expression and/or proteolytic processes regulated by rhomboids. Yet, it cannot be ruled out that some of these proteins are contaminants from membranous origin. Differential proteins with predicted SP or TMDs that may have been released to the cytoplasm because of rhomboid activity were identified. The list includes Q2YLG0 Leu/Ile/Val-binding protein homolog 2 (SP); Q2YRV5 31 kDa immunogenic protein (SP); Q2YJW2 Nitrous-oxide reductase (NosZ) (SP); Q2YJU8 Copper-containing nitrite reductase (SP); Q2YM85 Cbb3-type cytochrome c oxidase unit (1 TMD); Q2YMF9 small uncharacterized protein (SP); Q2YPP2 DUF1775 domain-containing protein (SP) and Q2YRS4 OmpW family protein (SP).

In the secretome, all differential proteins with SP or single TMD were underrepresented in Δ*rhoX*: a putative lytic murein transglycosylase, OmpW family protein, nitrous-oxide reductase NosZ and Cbb3-type cytochrome c oxidase subunit involved in the oxidative phosphorylation pathway.

Finally, rhomboid in *B. abortus* might target multiprotein membrane complexes participating in adaptation to low oxygen conditions by a yet unidentified mechanism, as evidenced by overexpression of wild type *rhomboid* in static cultures. We assume rhomboid activity might be tightly regulated and this phenotype is only observed after gene overexpression. A few years ago, a study demonstrated that in respiration at low oxygen tension, the two-component systems PrrBA and NtrYX coordinately regulate the expression of denitrification and high-affinity cytochrome oxidase genes [79]. Further studies are needed to determine if denitrification enzymes like NarG, NarH, NosZ and NorC, and high-affinity cyto-chrome cbb3 oxidase identified by the proteomics approach are rhomboid substrates. Also of interest is to determine whether *B. abortus* rhomboid targets orphan proteins to mediate quality control of respiratory protein complexes such as already described in *S. sonnei* [20].

## Figures and Tables

**Table 1:** Plasmids and bacterial strains used in this study.

Strain or Plasmid	Characteristics (*)	Reference or Source
*B. abortus* Strains		
2308	Wild type, smooth, virulent, Nal^r^	Laboratory stock
Δ*rhoX*	*Rhomboid* deletion mutant, Nal^r^	This work
Δ*rhoX* + pLF_*rhoX*	Rhomboid deletion mutant containing pLF_*rho* wild type, Amp^r^	This work
ΔrhoX + pLF_*rhoX*_S153A	*Rhomboid* deletion mutant containing pLF_*rho* S153A mutant, Amp^r^	This work
*E. coli* strains		
K12-DH5α (F’Iq)	Host strain used for cloning	Invitrogen
S17.1 (λpir)	Host strain used for conjugation	[31]
Plasmids		
pK18 *mob-sacB*	Cloning vector for unmarked gene deletion	[32]
pBBR1 MCS-2	Broad-host-range cloning vector, Kan^r^	[33]
pBBR2-*gurken*	pBBR1 MCS-2 carrying chimera *gurken* gene from pKS505, Km^r^	This work
pBBR2-*tatA*	pBBR1 MCS-2 carrying chimera *tatA* gene from pKS508, Km^r^	This work
pKS505	pKS29 encoding Gurken TMD, Amp^r^	[14]
pKS508	pKS29 encoding TatA (aa1–50), Amp^r^	[14]
pLF	Cloning vector (pBBR1-MCS4-3xFlag), Amp^r^	[34]
pLF_*rho*	pLF encoding rhomboid wild type, Amp^r^	This work
pLF_*rho* S153A	pLF encoding rhomboid S153A mutant, Amp^r^	This work
pTrc-EYFP	pBBR1-MCS4 derived, encoding enhancing yellow fluorescent protein under trc promoter, Amp^r^	[35]
pTrc-*rho*_EYFP	pTrc-EYFP derived, encoding rhomboid fused to *eyfp*, Amp^r^	This work

(*) Amp^r^, ampicillin resistance; Nal^r^, nalidixic acid resistance; Km^r^, kanamycin resistance.

**Table 2:** List of proteins differentially represented between *B. abortus* wild type and *rhomboid* mutant detected in TSB and RPMI-grown bacteria.

Uniprot Entry	*B. abortus* ORF	Protein
Q2YJW2	nosZ BAB2_0928	Nitrous-oxide reductase NosZ (N2O reductase)
Q2YLC1	BAB2_0280	Shikimate kinase: ATP/GTP-binding site motif A (P-loop):ABC transporter: AAA ATPase
Q2YM85	BAB1_0389	Cbb3-type cytochrome c oxidase subunit
Q2YP67	BAB1_0238	Bacterial extracellular solute-binding protein, family 1
Q2YP88	BAB1_0248	Mandelate racemase/muconate lactonizing enzyme
Q2YP89	BAB1_0247	ATP/GTP-binding site motif A (P-loop): Fumarylacetoacetate (FAA) hydrolase
Q2YP90	BAB1_0246	Short-chain dehydrogenase/reductase SDR: Glucose/ribitol dehydrogenase
Q2YP92	BAB1_0244	Oxidoreductase, N-terminal
Q2YP95	BAB1_0241	ATP/GTP-binding site motif A (P-loop):ABC transporter: AAA ATPase
Q2YRS4	BAB1_1579	OmpW family

## Data Availability

Mass spectrometry raw data are provided as Supplementary Table S1.

## Supplementary Materials

The following supporting information can be downloaded at: https://www.mdpi.com/article/10.3390/microorganisms10010114/s1: Table S1: mass spectrometry raw data, Table S2: List of proteins differentially represented in *Brucella abortus* wild type and *rhomboid* mutant.
