# Supplementary material for: Brucella abortus Encodes an Active Rhomboid Protease: Proteome Response after Rhomboid Gene Deletion
Source: Microorganisms. 2022 Jan 6;10(1):114. doi: 10.3390/microorganisms10010114 (PMC8778405; doi:10.3390/microorganisms10010114)
Supplement: Supplementary file 1 [file microorganisms-10-00114-s001.zip › microorganisms-10-00114-s001/Table S1.pdf]

**Table S1. List of proteins differentially represented in *Brucella abortus* wild type and *rhomboid* mutant**. Three biological replicates from 2308 and Δ*rhoX* strains were submitted to a Student's t-test. Proteins with *p*-values ≤ 0.05 were considered significant. TMD: number of transmembrane segments (predicted by ** TMHMM Server v. 2.0 (http://www.cbs.dtu.dk/services/TMHMM/). SP*: general translocation signal peptide (predicted by *signalP 5.0 (http://www.cbs.dtu.dk/services/SignalP/). TAT: Twin arginine translocation signal peptide. Subcellular localization was predicted according to Psortb (Psortb https://www.psort.org/psortb/index.html).

| **Increased in *B. abortus rhomboid* membrane<br>TSB** | | | | | |
|---|---|---|---|---|---|
| **Uniprot Entry** | **Gene** | **Protein** | **SP*** | **TMD**** | **Localization_Psortb***** |
| **Q2YJ09** | BAB2_0330 | Sarcosine oxidase beta subunit | -- | -- | Cytoplasmic |
| **Q2YJ13** | BAB2_0323<br>*nagB* | Sugar isomerase (SIS) | -- | -- | Cytoplasmic |
| **Q2YJ15** | BAB2_0328 | Uncharacterized protein containing DUF1338 domain | -- | -- | Cytoplasmic |
| **Q2YJ16** | BAB2_0327 | Aldehyde dehydrogenase | -- | -- | Cytoplasmic |
| **Q2YJ39** | BAB2_0428 | ATP/GTP-binding site motif A (P-loop):ABC transporter:AAA ATPase | -- | -- | Cytoplasmic Membrane |
| **Q2YJM8** | BAB2_1021<br>*cls* | Phospholipase D/Transphosphatidylase (Cardiolipin synthase) | -- | 2<br>(4-26)<br>(39-61) | Cytoplasmic Membrane |
| **Q2YKU9** | BAB2_0547 | Bacterial extracellular solute-binding protein, family 1 (probable sugar-binding periplasmic protein) | yes | -- | Periplasmic |
| **Q2YKW2** | BAB2_0532<br>*ahpD* | Alkyl hydroperoxide reductase AhpD | -- | -- | Unknown |
| **Q2YLB2** | BAB2_0289 | Pyruvate decarboxylase | -- | -- | Cytoplasmic |
| **Q2YLC0** | BAB2_0281 | ATP/GTP-binding site motif A (P-loop):ABC transporter:AAA ATPase (branched-chain amino acid ABC transporter, ATP-binfing protein) | -- | -- | Cytoplasmic Membrane |
| **Q2YLC1** | BAB2_0280 | Shikimate kinase:ATP/GTP-binding site motif A (P-loop):ABC transporter:AAA ATPase (branched-chain amino acid ABC transporter, ATP-binfing protein) | -- | -- | Unknown (This protein may have multiple localization sites) |
| **Q2YLG0; Q2YLF8** | BAB1_1792; BAB1_1794 | Leu/Ile/Val-binding protein homolog 2; Leu/Ile/Val-binding protein homolog 1 | yes | -- | Periplasmic; Periplasmic |
| **Q2YLI9** | BAB1_1849<br>*rsfS* | Ribosomal silencing factor RsfS | -- | -- | Cytoplasmic |
| **Q2YLP1** | BAB1_1869 | Uncharacterized protein containing DUF4167 domain | -- | -- | Unknown |
| **Q2YLZ7** | BAB1_1438 | Uncharacterized protein | -- | -- | Cytoplasmic |
| **Q2YM25** | BAB1_1499 | DNA primase | -- | -- | Cytoplasmic |

| | | | | | |
|---|---|---|---|---|---|
| | *dnaG* | | | | |
| **Q2YMH3** | BAB1_0476 *cfa* | SAM (And some other nucleotide) binding motif:Generic methyltransferase:Cyclopropane-fatty-acyl-phospholipid synthase | -- | -- | Cytoplasmic |
| **Q2YNA5** | BAB1_0777 *parC* | DNA topoisomerase 4 subunit A | -- | -- | Cytoplasmic (This protein may have multiple localization sites). |
| **Q2YPB7** | BAB1_0286 | Uncharacterized protein | -- | -- | Cytoplasmic |
| **Q2YPH7** | BAB1_0327 | Polyphosphate kinase | -- | -- | Cytoplasmic |
| **Q2YQB7** | BAB1_1649 *rbsC-2* | Bacterial inner-membrane translocator:Voltage-dependent potassium channel | -- | 8 | Cytoplasmic Membrane |
| **Q2YR09** | BAB1_2065 *rho* | Transcription termination factor Rho | -- | -- | Cytoplasmic |
| **Q2YRV5** | BAB1_1216 | 31kDa immunogenic protein that belongs to the TAXI family of the substrate-binding proteins | yes | -- | Unknown |
| **Q2YRB0** | BAB1_1240 *rplF* | 50S ribosomal protein L6 | -- | -- | Cytoplasmic |
| **Increased in *B. abortus rhomboid* cytoplasm TSB** | | | | | |
| **Q2YJ15** | BAB2_0328 | Uncharacterized protein containing DUF1338 domain | | -- | Cytoplasmic |
| **Q2YJ16** | BAB2_0327 | Aldehyde dehydrogenase | | -- | Cytoplasmic |
| **Q2YJ40** | BAB2_0427 | ABC transporter, periplasmic substrate-binding protein | yes (TAT) | -- | Unknown |
| **Q2YJV3** | BAB2_0938 | Periplasmic binding protein/LacI transcriptional regulator | yes | -- | Unknown (This protein may have multiple localization sites). |
| **Q2YLB9** | BAB2_0282 | Leu/Ile/Val-binding protein family | yes | -- | Unknown |
| **Q2YLC0** | BAB2_0281 | ATP/GTP-binding site motif A (P-loop):ABC transporter:AAA ATPase (branched-chain amino acid ABC transporter, ATP-binfing protein) | | -- | Cytoplasmic Membrane |
| **Q2YLG0** | BAB1_1792 | Leu/Ile/Val-binding protein homolog 2 | yes | -- | Periplasmic |
| **Q2YLG9** | BAB1_1827 | ATP/GTP-binding site motif A (P-loop):Bacterial NAD-glutamate dehydrogenase | | -- | Cytoplasmic Membrane |
| **Q2YLP1** | BAB1_1869 | Uncharacterized protein containing DUF4167 domain | | -- | Unknown |
| **Q2YMQ4** | *rbsB-1* BAB1_0567 | Periplasmic binding protein/LacI transcriptional regulator | yes | -- | Periplasmic |

| | | | | | |
|---|---|---|---|---|---|
| **Q2YNH9** | *phnM* BAB1_0879 | Amidohydrolase | | -- | Cytoplasmic |
| **Q2YPZ4** | BAB1_1109 | Acyl-CoA dehydrogenase:Acyl-CoA dehydrogenase, C-terminal:Acyl-CoA dehydrogenase, central domain:Acyl-CoA dehydrogenase, N-te | | -- | Cytoplasmic |
| **Q2YQE3** | BAB1_1362 | Periplasmic binding protein/LacI transcriptional regulator | yes | -- | Unknown |
| **Q2YQK0** | BAB1_1333 | Acyl-CoA dehydrogenase:Acyl-CoA dehydrogenase, C-terminal:Acyl-CoA dehydrogenase, central domain:Acyl-CoA dehydrogenase, N-te | | -- | Cytoplasmic |
| **Q2YRV5** | BAB1_1216 | 31kDa immunogenic protein that belongs to the TAXI family of the substrate-binding proteins | yes | -- | Unknown |
| **Increased in *B. abortus rhomboid* cytoplasm RPMI** | | | | | |
| **Q2YLC1** | BAB2_0280 | Shikimate kinase:ATP/GTP-binding site motif A (P-loop):ABC transporter:AAA ATPase (branched-chain amino acid ABC transporter, ATP-binfing protein) | -- | -- | Unknown |
| **Q2YQ38** | BAB1_1054 | ATP/GTP-binding site motif A (P-loop):YcjX-like protein | -- | -- | Cytoplasmic |
| **Q2YR39** | *accA* BAB1_2033 | Acetyl-coenzyme A carboxylase carboxyl transferase subunit alpha | -- | -- | Cytoplasmic |
| **Q2YQW8** | *accD* BAB1_2109 | Acetyl-coenzyme A carboxylase carboxyl transferase subunit beta | -- | -- | Cytoplasmic |
| **Q2YP76** | BAB1_0226 | Substrate-binding region of ABC-type glycine betaine transport system | yes | -- | Cytoplasmic membrane |
| **Increased in *B. abortus wild type* membrane TSB** | | | | | |
| **Q2YIK8** | BAB2_0233 *nosA* | Heme transporter BhuA | yes | -- | Outer Membrane |
| **Q2YJT6** | BAB2_0955 *norC* | Nitric oxide reductase subunit C (NorC) | -- | 1 (13-35) | Cytoplasmic Membrane |
| **Q2YJT8** | BAB2_0953 *norQ* | Chaperonin clpA/B:ATP/GTP-binding site motif A (P-loop) | -- | -- | Cytoplasmic |
| **Q2YJX5** | BAB2_0915 | Ubiquinone biosynthesis protein UbiV | -- | -- | Cytoplasmic |
| **Q2YJX6** | BAB2_0914 | Ubiquinone biosynthesis protein UbiU | -- | -- | Cytoplasmic |
| Q2YJY0 | BAB2_0908 | Parvulin-like Peptidylprolyl isomerase | -- | -- | Cytoplasmic |
| **Q2YJY3** | BAB2_0905 *narH* | Cytochrome c heme-binding site:4Fe-4S ferredoxin, iron-sulfur binding domain:Nitrate reductase, beta subunit | -- | -- | Cytoplasmic Membrane |

| | | | | | |
|---|---|---|---|---|---|
| **Q2YJY4** | *narG* BAB2_0904 | Nitrate reductase, alpha subunit:Prokaryotic molybdopterin oxidoreductase:Molybdopterin oxidoreductase:Molydopterin dinucleot | -- | -- | Cytoplasmic Membrane |
| **Q2YJZ9** | *minD* BAB2_0883 | Cell division inhibitor MinD | -- | -- | Cytoplasmic (This protein may have multiple localization sites.) |
| **Q2YK88** | BAB2_0777 | Bacterial regulatory protein, GntR family | -- | -- | Cytoplasmic |
| **Q2YKF1** | BAB2_0709 | DNA translocase FtsK | -- | -- | Cytoplasmic Membrane |
| **Q2YKR8** | BAB2_0582 *ugpC* | sn-glycerol-3-phosphate import ATP-binding protein UgpC | -- | -- | Cytoplasmic Membrane |
| **Q2YKW3** | BAB2_0531 *ahpC* | Alkyl hydroperoxide reductase C | -- | -- | Cytoplasmic |
| **Q2YLP9** | BAB1_1903 *sdhC* | Succinate dehydrogenase cytochrome b556 subunit | -- | 3 (29-51) (66-88) (109-131) | Cytoplasmic Membrane |
| **Q2YM73** | BAB1_1294 | Aminotransferase | -- | -- | Cytoplasmic |
| **Q2YN08** | BAB1_0675 | Coproporphyrinogen-III oxidase | -- | -- | Cytoplasmic |
| **Q2YNS1** | BAB1_0964 | Uncharacterized protein | -- | -- | Cytoplasmic |
| **Q2YP16** | BAB1_0154 | LPS export ABC transporter periplasmic protein LptC | -- | 1 (25-47) | Unknown |
| **Q2YP67** | BAB1_0238 | Bacterial extracellular solute-binding protein, family 1 | yes | -- | Unknown |
| **Q2YP89** | BAB1_0247 | ATP/GTP-binding site motif A (P-loop): Fumarylacetoacetate (FAA) hydrolase | -- | -- | Cytoplasmic |
| **Q2YP90** | BAB1_0246 | Short-chain dehydrogenase/reductase SDR:Glucose/ribitol dehydrogenase | -- | -- | Cytoplasmic |
| **Q2YP94** | BAB1_0242 | Mandelate racemase/muconate lactonizing enzyme | -- | -- | Cytoplasmic |
| **Q2YP95** | BAB1_0241 | ATP/GTP-binding site motif A (P-loop):ABC transporter:AAA ATPase | -- | -- | Cytoplasmic Membrane |
| **Q2YQC8** | BAB1_1371 | Gram-negative bacterial RTX secretion protein D: Secretion protein HlyD | yes | -- | Cytoplasmic Membrane |
| **Q2YQQ0** | BAB1_2182 | Cytosol aminopeptidase | -- | -- | Cytoplasmic |
| **Increased in *B. abortus wild type* membrane RPMI** | | | | | |
| **Q2YRS4** | BAB1_1579 | OmpW family protein | yes | -- | Outer membrane |
| **Q2YPQ2** | BAB1_0038 | Ubiquinol oxidase subunit 2 | -- | 3 | Cytoplasmic membrane |

| | | | | | |
|---|---|---|---|---|---|
| **Q2YP95** | BAB1_0241 | ATP/GTP-binding site motif A (P-loop):ABC transporter:AAA ATPase | -- | -- | Cytoplasmic membrane |
| **Increased in *B. abortus wild type* cytoplasm TSB** | | | | | |
| **Q2YIH3** | BAB2_0211 | Uncharacterized protein | | -- | Unknown |
| **Q2YJT8** | BAB2_0953 | Chaperonin clpA/B:ATP/GTP-binding site motif A (P-loop) | | -- | Cytoplasmic |
| **Q2YJU8** | BAB2_0943 | Copper-containing nitrite reductase | yes (TAT) | -- | Periplasmic |
| **Q2YJW2** | *nosZ* BAB2_0928 | Nitrous-oxide reductase (N$_2$O reductase) | yes (TAT) | -- | Periplasmic |
| **Q2YJX6** | BAB2_0914 | Ubiquinone biosynthesis protein UbiU | | -- | Cytoplasmic |
| **Q2YJY3** | *narH* BAB2_0905 | Cytochrome c heme-binding site:4Fe-4S ferredoxin, iron-sulfur binding domain:Nitrate reductase, beta subunit | | -- | Cytoplasmic Membrane |
| **Q2YJZ9** | *minD* BAB2_0883 | Cell division inhibitor MinD | | -- | Cytoplasmic (This protein may have multiple localization sites.) |
| **Q2YKF1** | BAB2_0709 | DNA translocase FtsK | | -- | Cytoplasmic Membrane |
| **Q2YKW3** | *ahpC* BAB2_0531 | Alkyl hydroperoxide reductase C | | -- | Cytoplasmic |
| **Q2YM85** | BAB1_0389 | Cbb3-type cytochrome c oxidase subunit | | 1 (32-54) | Unknown |
| **Q2YMF9** | BAB1_0453 | Uncharacterized protein | yes | -- | Unknown |
| **Q2YN08** | BAB1_0675 | Coproporphyrinogen-III oxidase | | -- | Cytoplasmic |
| **Q2YN78** | BAB1_0740 | Uncharacterized protein | | -- | Cytoplasmic |
| **Q2YNC7** | *nuoC* BAB1_0824 | NADH-quinone oxidoreductase subunit C (NADH dehydrogenase I subunit C) | | -- | Cytoplasmic |
| **Q2YNJ4** | BAB1_0863 | Uncharacterized protein | | -- | Unknown |
| **Q2YNT6** | BAB1_0974 | GBBH-like_N domain-containing protein | | -- | Unknown |
| **Q2YNU1** | BAB1_0090 | Aconitate hydratase (Aconitase) | | -- | Cytoplasmic |
| **Q2YNW5** | BAB1_0100 | Transcriptional regulator, putative | | -- | Unknown |
| **Q2YP18** | *ihfB* BAB1_0152 | Integration host factor subunit beta (IHF-beta) | | -- | Cytoplasmic |
| **Q2YP66** | BAB1_0204 | Zinc-containing alcohol dehydrogenase superfamily:Zinc-containing alcohol dehydrogenase | | -- | Cytoplasmic |
| **Q2YP67** | BAB1_0238 | Bacterial extracellular solute-binding protein, family 1 | yes | -- | Unknown |

| | | | | | |
|---|---|---|---|---|---|
| **Q2YP69** | BAB1_0236 | Amidohydrolase 2 | | -- | Cytoplasmic |
| **Q2YP88** | BAB1_0248 | L-fuconate dehydratase | | -- | Cytoplasmic |
| **Q2YP89** | BAB1_0247 | ATP/GTP-binding site motif A (P-loop): Fumarylacetoacetate (FAA) hydrolase | | -- | Cytoplasmic |
| **Q2YP90** | BAB1_0246 | Short-chain dehydrogenase/reductase SDR:Glucose/ribitol dehydrogenase | | -- | Cytoplasmic |
| **Q2YP92** | BAB1_0244 | Oxidoreductase, N-terminal | | -- | Cytoplasmic |
| **Q2YP94** | BAB1_0242 | Mandelate racemase/muconate lactonizing enzyme | | -- | Cytoplasmic |
| **Q2YPE0** | BAB1_0295 | HTH cro/C1-type domain-containing protein | | -- | Unknown |
| **Q2YPP2** | BAB1_0051 | Uncharacterized protein containing DUF1775 | yes | -- | Unknown |
| **Q2YQA8** | BAB1_1610 | N-acetyltransferase domain-containing protein | | -- | Unknown |
| **Q2YQG8** | BAB1_1280 | Uncharacterized protein | | -- | Unknown |
| **Q2YQQ4** | BAB1_2178 | TrkA potassium uptake protein:D-isomer specific 2-hydroxyacid dehydrogenase, catalytic domain:D-isomer specific 2-hydroxyacid | | -- | Cytoplasmic |
| **Q2YRG3** | BAB1_1297 | Uncharacterized protein | | -- | Cytoplasmic |
| **Q2YRS4** | BAB1_1579 | OmpW family protein | yes | -- | Outer Membrane |
| **Increased in *B. abortus wild type* cytoplasm RPMI** | | | | | |
| **Q2YJ50** | *vjbR* BAB2_0118 | HTH-type quorum sensing-dependent transcriptional regulator VjbR | -- | -- | Unknown |
| **Q2YLR4** | *dapF* BAB1_1932 | Diaminopimelate epimerase | -- | -- | Unknown |
| **Q2YP88** | BAB1_0248 | L-fuconate dehydratase | -- | -- | Cytoplasmic |
| **Q2YP92** | BAB1_0244 | Oxidoreductase, N-terminal | -- | -- | Cytoplasmic |
| **Q2YJZ3** | *nrdE* BAB2_0889 | Ribonucleoside-diphosphate reductase | -- | -- | Cytoplasmic |
| **Q2YQB2** | *mnmA* BAB1_1607 | tRNA-specific 2-thiouridylase MnmA | -- | -- | Cytoplasmic |
| **Q2YP95** | BAB1_0241 | ATP/GTP-binding site motif A (P-loop): ABC transporter:AAA ATPase | -- | -- | Cytoplasmic membrane |
| **Q2YP89** | BAB1_0247 | ATP/GTP-binding site motif A (P-loop): Fumarylacetoacetate (FAA) hydrolase | -- | -- | Cytoplasmic |
| **Q2YLG4** | *ispG* BAB1_1788 | 4-hydroxy-3-methylbut-2-en-1-yl diphosphate synthase (flavodoxin) | yes (TAT) | -- | Cytoplasmic |
| **Q2YRK6** | BAB1_1662 | Glutamyl-tRNA synthetase, class Ic:Aminoacyl-tRNA synthetase, class I | -- | -- | Cytoplasmic |

| | | | | | |
|---|---|---|---|---|---|
| **Q2YP67** | BAB1_0238 | Bacterial extracellular solute-binding protein, family 1 | yes | -- | Unknown |
| **Q2YP90** | BAB1_0246 | Short-chain dehydrogenase/reductase SDR:Glucose/ribitol dehydrogenase | -- | -- | Cytoplasmic |
| **Q2YNS3** | BAB1_0962 | L-isoaspartyl protein carboxyl methyltransferase | -- | -- | Cytoplasmic |
| **Q2YL51** | *leuD* BAB2_0353 | 3-isopropylmalate dehydratase small subunit | -- | -- | Cytoplasmic |
| **Q2YQA4** | *ctrA* BAB1_1614 | Cell cycle response regulator CtrA | -- | -- | Cytoplasmic |
| **Q2YJZ4** | *nrdF* BAB2_0888 | Ribonucleoside-diphosphate reductase subunit beta | -- | -- | Cytoplasmic |
| **Increased in *B. abortus rhomboid* secretome** | | | | | |
| **Q2YLF3** | BAB1_1800 | 4-hydroxyproline epimerase | -- | -- | Cytoplasmic |
| **Q2YJ23** | BAB2_0292 | Sugar transporter | -- | -- | Cytoplasmic |
| **Q2YM05** | BAB1_1253 | 50S ribosomal protein L23 | -- | -- | Cytoplasmic |
| **Increased in *B. abortus wild type* secretome** | | | | | |
| **Q2YIT4** | BAB2_0069 | Putative peptidoglycan binding domain 1 (lytic murein transglycosylase) | yes | -- | Cytoplasmic membrane |
| **Q2YLX8** | BAB1_1549 | Ribose-phosphate pyrophosphokinase | -- | -- | Cytoplasmic |
| **Q2YNS2** | BAB1_0963 | Outer membrane efflux protein | yes | -- | Outer membrane |
| **Q2YQA6** | BAB1_1612 | Uncharacterized protein (EipA) | yes | 1 | Cytoplasmic membrane |
| **Q2YRS4** | BAB1_1579 | OmpW family protein | yes | -- | Outer membrane |
| **Q2YQ61** | BAB1_1029 | Uncharacterized protein containing DUF2059 | yes | -- | Unknown |
| **Q2YN61** | BAB1_0729 | Uncharacterized protein | yes | -- | Unknown |
| **Q2YJW2** | BAB2_0928 *nosZ* | Nitrous-oxide reductase | yes (TAT) | -- | Periplasmic |
| **Q2YM03** | BAB1_1255 | 50S ribosomal protein L3 | -- | -- | Cytoplasmic |
| **Q2YMS0** | BAB1_0587 | Uncharacterized protein | -- | -- | Unknown |
| **Q2YM85** | BAB1_0389 | Cbb3-type cytochrome c oxidase subunit | -- | 1 (32-54) | Unknown |
